# Medical Applications and Toxicities of Gallium Compounds

**DOI:** 10.3390/ijerph7052337

**Published:** 2010-05-10

**Authors:** Christopher R. Chitambar

**Affiliations:** Division of Neoplastic Diseases, Department of Medicine, Medical College of Wisconsin, 9200 West Wisconsin Avenue, Milwaukee, WI 53226, USA; E-Mail: chitambr@mcw.edu; Tel.: +1-414-805-4600; Fax: +1-414-805-4606

**Keywords:** Gallium, iron, iron proteins, electronics, semi-conductors, calcium and bone metabolism, cancer therapeutics, toxicities

## Abstract

Over the past two to three decades, gallium compounds have gained importance in the fields of medicine and electronics. In clinical medicine, radioactive gallium and stable gallium nitrate are used as diagnostic and therapeutic agents in cancer and disorders of calcium and bone metabolism. In addition, gallium compounds have displayed anti-inflammatory and immunosuppressive activity in animal models of human disease while more recent studies have shown that gallium compounds may function as antimicrobial agents against certain pathogens. In a totally different realm, the chemical properties of gallium arsenide have led to its use in the semiconductor industry. Gallium compounds, whether used medically or in the electronics field, have toxicities. Patients receiving gallium nitrate for the treatment of various diseases may benefit from such therapy, but knowledge of the therapeutic index of this drug is necessary to avoid clinical toxicities. Animals exposed to gallium arsenide display toxicities in certain organ systems suggesting that environmental risks may exist for individuals exposed to this compound in the workplace. Although the arsenic moiety of gallium arsenide appears to be mainly responsible for its pulmonary toxicity, gallium may contribute to some of the detrimental effects in other organs. The use of older and newer gallium compounds in clinical medicine may be advanced by a better understanding of their mechanisms of action, drug resistance, pharmacology, and side-effects. This review will discuss the medical applications of gallium and its mechanisms of action, the newer gallium compounds and future directions for development, and the toxicities of gallium compounds in current use.

## 1. Introduction

Gallium is a group IIIA metal, atomic number 31 in the periodic table of elements. First discovered in 1875 by Paul-Emile Lecoq de Boisbaudran in France, the name of this metal appears to be derived from “Gallia”, the Latin word for France. However, it is also thought that perhaps the discoverer Lecoq named the metal after himself by calling it gallum, the Latin translation for a cock. Gallium is present at a concentration of 5–15 mg/kg in the earth’s crust and is obtained as a byproduct from the extraction of aluminum and zinc ores [1]. It has a shiny, silvery-white color, with a melting temperature of 28.7646 °C (85.5763 °F); it is one of the few metals that is near-liquid at room temperature and can melt when held in the hand. Although gallium has no known physiologic function in the human body, certain of its characteristics enable it to interact with cellular processes and biologically important proteins, especially those of iron metabolism. This has led to the development of certain gallium compounds as diagnostic and therapeutic agents in medicine especially in the areas of metabolic bone disease, cancer, and infectious disease. The discovery that gallium displayed semiconducting properties led to its development as gallium arsenide for use in the electronics industry [2]. In the USA, more than 95% of gallium consumed is for optoelectronic devices and integrated circuits [1]. The application of gallium in medicine raises questions about the pharmacology, clinical efficacy, and potential side-effects of gallium compounds as drugs. The use of gallium arsenide in the electronics industry raises questions about the potential risks of exposure to this compound as an environmental toxin. The reader is also referred to earlier reviews on gallium [3–8]. The present review will provide an update of the medical applications of gallium and the toxicities of gallium compounds in medicine and the electronics industry.

## 2. Medical Applications of Gallium Compounds

### 2.1. Radiogallium Compounds as Tumor Imaging Agents

Early studies demonstrated that radioactive gallium (^67^Ga citrate) injected into tumor-bearing animals localized in malignant cells [9]. This observation led to the development of the ^67^Ga scan for the detection of malignant tumors in patients [10]. Over the past two decades, ^67^Ga scanning has been used most frequently in patients with Hodgkin’s and non-Hodgkin’s lymphomas to detect residual disease or disease that has relapsed following treatment with chemotherapy or radiotherapy [11–14]. The level of ^67^Ga incorporation into lymphoma cells reflects the metabolic activity of the tumor and is indicative of the presence of viable malignant cells. Hence, a positive ^67^Ga scan after treatment of lymphoma generally indicates the presence of residual malignancy and the need for further therapy. The intensity of ^67^Ga uptake by malignancies such as lymphoma correlates with their proliferative rate; ^67^Ga scanning thus provides a valuable insight into the aggressiveness of a tumor [12]. Gallium may also be taken up by macrophages and thus, areas of inflammation may show ^67^Ga-positivity on the ^67^Ga scan. Recently, the ^67^Ga scan in lymphoma has been replaced by the Positron Emission Tomography (PET) scan which is based on the uptake of ^18^F-fluorodeoxyglucose (FDG) by tumors [15]. However, the ^67^Ga scan remains an important diagnostic modality in geographical locations where the ^18^F-FDG PET scan may not be available.

Although tumor imaging with ^18^F-FDG PET is now being increasingly used for a variety of malignancies, it has its limitations. Tumors with a low growth rate such as hepatocellular carcinoma, neuroendocrine tumors, prostate cancer, and indolent (slow-growing) lymphomas may not take up FDG. Conversely, tumors surrounded by or adjacent to normal tissue with high metabolic activity (such as the brain) may be difficult to assess by ^18^F-FDG PET because of high background FDG uptake by the normal tissue (*i.e.*, a low ratio of tumor/normal tissue FDG uptake). As a result of these limitations, there has been considerable interest in the development of ^68^Ga-labeled radiopharmaceuticals for molecular tumor imaging (reviewed in references 16 and 17). This gallium isotope has a short half-life of approximately 68 minutes which is an advantage to its clinical use. Peptides labeled with radiometals are showing promising clinical activity as imaging agents for the diagnosis and management of neuroendocrine tumors, neural crest tumors (pheochromocytomas and paragangliomas), meningiomas, and prostate cancer. Somatostatin, which targets the somatostatin receptor subtypes present in increased numbers on these cells, has been the most developed model for synthetic peptides. The somatostatin analogue D-Phe^1^-Tyr^3^-octreotide (TOC) coupled to the bifunctional chelate DOTA (1,4,7,10-tetraazacyclododecane-1,4,7,10-teraacetic acid) labeled with ^68^Ga (^68^Ga-DOTATOC) has been used with great success for the imaging of neuroendocrine tumors in patients. In other studies, ^68^Ga-DOTA-D-Phe^1^-Tyr3-Thr8-octreotide (^68^Ga-DOTA-TATE) PET was used to image the somatostatin receptor in recurrent pheochromocytoma and meningiomas in patients and in the latter disease led to changes in radiotherapy planning. Imaging of neuroendocrine tumors with another somatostatin analogue, ^68^Ga-DOTANOC has also shown promise [18], while ^68^Ga-DOTA-alpha melanocyte-stimulating hormone analog has been used for imaging metastases in melanoma [19]. In addition ^68^Ga labeled-DOTA coupled to bombesin (^68^Ga-DOTA-BOM) PET has been used to diagnose prostate cancer expressing the bombesin receptor, while ^67^Ga- and ^68^Ga-deferoxamine (DFO)-folate have been used to image tumors that express high levels of cell surface folate receptors in a tumor xenograft model [20,21].

### 2.2. Antineoplastic Activity of Gallium Nitrate in Cancer Treatment

The ability of ^67^Ga to localize in tumor cells prompted investigators at the National Cancer Institute (NCI) to investigate the antineoplastic activities of salts of the group IIIa metals aluminum, gallium, indium, and thallium in tumor-bearing CDF1 mice and Sprague-Dawley rats [22]. Of these metal compounds, gallium nitrate proved to be both highly effective in suppressing the growth of subcutaneously implanted tumors and least toxic. As a result, it was advanced to NCI investigational drug status (NSC 15200) for assessment of toxicity and antitumor efficacy in Phase 1 and Phase 2 clinical trials [23]. In those clinical studies, gallium nitrate was administered by two different schedules: a brief (15–30 minutes) intravenous infusion or a continuous intravenous infusion given over 24 hours for 5 to 7 days.

While the clinical efficacy of continuous infusion gallium nitrate has been examined in a number of different malignancies, this drug has displayed its strongest antineoplastic activity in the treatment of non-Hodgkin’s lymphoma and bladder cancer. At least 4 separate clinical trials have demonstrated the anti-lymphoma activity of gallium nitrate administered as a single agent [24–27], while 3 additional trials have used it in combination with other antineoplastic agents to improve treatment outcome [28–30]. In all these studies, gallium nitrate was assessed in patients with relapsed non-Hodgkin’s lymphoma that failed to respond to many conventional chemotherapeutic drugs and also to stem cell transplantation. Overall, studies indicate that approximately 30% of patients with relapsed lymphomas will responded to treatment with gallium nitrate. Interestingly, aggressive large cell and mantle cell lymphomas appear to be more responsive to gallium nitrate than other lymphoma subtypes [27]. The activity of gallium nitrate in advanced bladder cancer has also been confirmed in several clinical studies. At least three studies have demonstrated an overall response in 17–63% of patients treated with single agent gallium nitrate [31–33], while three additional studies using gallium nitrate in combination with other drugs have shown overall response rates of 12–67% [34–36]. The 12% response rate was seen in a study that combined gallium nitrate with fluorouracil [36]. This low response is surprising given the higher responses seen in other studies; it raises the question as to whether this was the result of drug antagonism between fluorouracil and gallium nitrate. An advantage of gallium nitrate is that unlike many chemotherapeutic drugs, it does not produce myelosuppression and can therefore be used in patients with low white blood cell or platelet counts. *In vitro*, gallium has shown to be synergistic with hydroxyurea, fludarabine, interferon-alpha, gemcitabine, and paclitaxel, suggesting that combination therapy with these agents may be fruitful [37–41]. Moreover, gallium nitrate does not appear to share cross-resistance with conventional chemotherapeutic drugs since clinical responses have been noted in patients who have failed to respond to multiple other drugs.

### 2.3. Effects of Gallium Nitrate on Malignancy-Associated Hypercalcemia and Bone Metabolism

Studies examining the antineoplastic activity of gallium nitrate revealed that a decrease in blood calcium levels occurred in a significant number of patients being treated with this agent [26]. Based on this observation, further investigations were pursued to examine the potential of gallium nitrate as a treatment for elevated blood calcium levels associated with cancer [42]. The bisphosphonate drugs have had an established role in the treatment of hypercalcemia and osteoporosis; randomized clinical trials were therefore conducted to compare the efficacy of gallium nitrate with the bisphosphonates etidronate and pamidronate in the treatment of hypercalcemia in cancer patients [43,44]. These studies demonstrated continuous infusion gallium nitrate (200 mg/m^2^/day for 5 days) to be superior to etidronate and at least as effective as pamidronate in acutely controlling elevated blood calcium levels. Gallium nitrate was also shown to be superior to calcitonin, a non-bisphosphonate drug used for treatment of hypercalcemia [45]. Based on its clinical efficacy, gallium nitrate (Ganite™) was approved by the Food and Drug Administration (FDA) for the treatment of cancer-associated hypercalcemia. Zoledronic acid, a newer generation bisphosphonate, has been shown to be more potent than pamidronate for treatment of hypercalcemia of malignancy [46]; however, randomized trials comparing gallium nitrate and zoledronic acid have not been conducted. Apart from its ability to control hypercalcemia, gallium nitrate has been shown to inhibit bone turnover and to decrease osteolysis in patients with multiple myeloma and in patients with bone metastases from a variety of different cancers [47,48]. Furthermore, administration of low, non-toxic doses of gallium nitrate to patients with Paget’s disease of bone, a disorder characterized by abnormal bone remodeling secondary to increased bone resorption by osteoclasts, led to a reduction in serum alkaline phosphatase, urinary hydroxyproline, and *N*-telopeptide, biochemical markers of bone turnover [49].

### 2.4. Potential Application of Gallium Compounds as Immunosuppressive and Anti-Inflammatory Agents

Evidence that environmental gallium per se may be immunosuppressive was provided by the studies of Betoulle *et al.* in which an aquatic system was used to show that that fish (carp) exposed to sublethal concentrations of gallium nitrate in the water for up to 96 hours displayed a significant reduction in immune parameters including immunoglobulin production, phagocyte killing ability, and blood leukocytes [50]. Several studies using *in vitro* and *in vivo* animal systems have shown that gallium compounds have immunosuppressive activity in animal models of autoimmune disease. Gallium nitrate has been shown to suppress experimental autoimmune encephalomyelitis and prevent adjuvant inflammatory arthritis through suppression of macrophage function and T-cells in rat models [51,52]. Other studies showed that gallium nitrate can suppress lupus and prevent cardiac allograft rejection in murine models [53,54]. Transferrin-gallium and gallium nitrate were shown to inhibit the mixed lymphocyte culture response and prolong the survival of mice with severe graft-versus-host disease in a murine bone marrow transplant model [55]. Despite these interesting preclinical observations, the immunomodulatory and anti-inflammatory properties of gallium appear to have not been investigated in rigorous clinical studies. Further investigations appear warranted to establish whether the results of these *in vitro* and animal studies are relevant to patients with inflammatory or autoimmune diseases. The potential effects of gallium on inflammation and the immune system should be kept in mind when gallium compounds are being used for the treatment of other conditions.

### 2.5. Pharmacology

Much of our understanding of gallium’s action on cells and its toxicity is derived from investigations conducted to evaluate its potential as a therapeutic agent. Insights into the pharmacology of gallium were provided by early studies which demonstrated that radioactive ^67^Ga citrate injected into rabbits was transported in the circulation exclusively bound to transferrin, the transport protein for iron [56]. Harris and Sephton showed that the uptake of radiogallium by malignant cell lines *in vitro* could be significantly enhanced by the addition of transferrin to the culture medium [57]; the clinical relevance of this finding was underscored by the studies of Vallabhajosula *et al.* who showed that in the blood radiogallium binds to transferrin and is transported to tumor tissue [56]. In this respect, the initial transport of gallium in the blood resembles that of iron. However, whereas gallium shares certain properties with ferric iron [Fe(III)] with respect to ionic radius and bonding, trivalent gallium unlike Fe cannot be reduced to a divalent state. Nonradioactive gallium binds avidly to both metal-binding sites on transferrin, but it does so with a 300-fold less affinity than ferric iron [58]. Under physiologic conditions, only about one-third of circulating transferrin is occupied by iron; hence, the metal-binding sites of the remaining transferrin molecules are available to bind and transport gallium as transferrin-gallium complexes. With higher levels of gallium in the circulation, transferrin binding is exceeded, and gallium likely circulates as gallate, Ga(OH)^4−^ [3].

Gallium pharmacokinetics have been examined in Phase-1 and -2 clinical trials in which gallium nitrate was administered to patients as a brief or a continuous intravenous infusion. When administered as an intravenous infusion over 30 minutes at doses ranging from 400–700 mg/m^2^ gallium nitrate, a biphasic gallium urinary excretion pattern with an α-phase half-life of 8.3–26 minutes and a β-phase half-life of 6.3–196 hours was noted [59]. The latter phase reflects its binding to transferrin in the circulation. Sixty-nine percent and 91% of the gallium dose administered was excreted in the urine during the first 24 and 48 hours, respectively [59]. Similar serum gallium disappearance curves were noted when higher concentrations of gallium nitrate (500–900 mg/m^2^) were administered by brief infusion. Hydration and co-administration of mannitol with gallium nitrate increased the amount of gallium excreted in the urine over the first hour [60]. In contrast to the peak serum levels of gallium attained when gallium nitrate is administered by brief infusion, administration of gallium nitrate by continuous intravenous infusion at its currently recommended dose of 200 mg/m^2^/day for 7 days results in a steady-state plasma gallium level of 0.9–1.9 μg/ml which decreases to 0.45–0.7 μg/ml at 4 days after stopping the drug. The continuous intravenous infusion schedule for gallium nitrate has much less toxicity than the brief infusion schedule and allows patients to receive twice the amount of gallium.

### 2.6. Mechanisms of Action

#### 2.6.1. Bone metabolism

The mechanisms of action of gallium nitrate on bone are distinct from those of bisphosphonates. Gallium, at non-toxic concentrations in a dose-dependent manner, accumulates in the metaphysis and diaphysis of bone at the interface of the organic (collagen) and the mineral components, thus altering crystal solubility and rendering bone more resistant to resorption (reviewed in reference 61). Significant increases in bone calcium content occur in gallium-treated bone which also makes bone less likely to be resorbed. In addition, gallium acts by blocking osteoclast activity without affecting the viability of these cells [61]. Osteoclasts play a critical role in bone resorption, modeling, and remodeling [62]. In the rat osteoclast-like cell line, gallium nitrate reduced vitamin D_3_-stimulated osteocalcin and osteopontin mRNA levels [63] and increased type 1 collagen and fibronectin expression in bone and fibroblast cells [64]. Collectively, studies indicate that while gallium nitrate is an effective drug for lowering pathologically elevated blood calcium levels in cancer, it may also have a broader application in diseases associated with increased bone loss such as osteoporosis, bone metastases, multiple myeloma, and Paget’s disease of the bone. Presently, gallium nitrate needs to be given to patients by continuous intravenous administration which is cumbersome and inconvenient. However, an oral formulation of this drug that appears promising is being advanced in early clinical trials [65].

#### 2.6.2. Antineoplastic activity

Several investigations have shown that gallium is taken up by various malignant cells *in vitro* through cell surface transferrin receptor-mediated endocytosis of Ga-transferrin complexes [66–69]. Evidence for a role for the transferrin receptor in gallium uptake was also provided by studies showing that the uptake of ^67^Ga by leukemic cells *in vitro* and by melanoma tumor in a rodent model could be blocked by monoclonal antibodies against the transferrin receptor [67,70]. In addition to transferrin receptor-facilitated uptake, a certain amount of gallium may be incorporated into cells through transferrin-independent mechanisms [67,69,71]. Hence, both transferrin receptor-dependent and -independent pathways for gallium uptake exist, and it is conceivable that their relative contributions to gallium uptake may vary with cell type.

The mechanisms of antineoplastic activity of gallium are complex and can be viewed as a two-step process in which the first step involves the targeting of gallium (as transferrin-gallium) to transferrin receptor-bearing tumor cells. Indeed, the high expression of transferrin receptors known to be present on lymphomas and bladder cancer cells may explain, in part, the preferential sensitivity of these malignancies to treatment with gallium nitrate [72–75]. In the second step, gallium perturbs cellular iron metabolism by interfering with transferrin receptor-mediated uptake of iron and with the endosomal release of iron from transferrin to the cytoplasm [76]. This leads to a condition of relative cellular iron deprivation and inhibition of the iron-dependent function of ribonucleotide reductase, the enzyme essential for deoxyribonucleotide synthesis [37]. Ribonucleotide reductase is a heterodimer protein consisting of R1 and R2 subunits; the R2 subunit contains a binuclear iron center and a tyrosyl free radical that are essential for enzyme function [77]. Perturbation of cellular iron homeostasis by gallium limits iron availability for R2 activity thus blocking ribonucleotide reductase activity and consequently DNA synthesis [37,78]. In addition, gallium has been shown to directly inhibit ribonucleotide reductase enzyme activity in cell lysates [79]; this indicates that gallium inhibits deoxyribonucleotide synthesis by dual mechanisms: a blockade of intracellular iron trafficking to the enzyme and a direct action on the enzyme that is independent of iron transport.

Recent studies have provided additional insights into the mechanisms of gallium-induced cell death in lymphoma. Gallium nitrate and gallium maltolate, a new gallium compound, induce apoptosis in human lymphoma cell lines through an intrinsic mitochondrial pathway that involves the activation of proapoptotic Bax, loss of mitochondrial membrane potential, release of cytochrome c from the mitochondria, and the activation of caspase-3 [80,81]. Exposure of human lymphoma CCRF-CEM cells to gallium nitrate results in the generation of reactive oxygen species (ROS) and a decrease in the GSH/GSSG ratio within 4 hours of incubation, long before any loss of cell viability occurs. This is followed by an induction of metallothionein-2A and heme oxygenase-1 expression which can be inhibited by the antioxidant N-acetyl cysteine [82]. In addition, gallium exposure leads to phosphorylation of p38 mitogen-activated protein kinase and the activation of Nrf-2, a transcription factor for heme oxygenase-1 [82]. These results suggest a model in which gallium nitrate-induced ROS production initially leads to a cytoprotective response (induction of metallothionein and heme oxygenase-2 production) and that cell death ensues when this protective response is overwhelmed. A protective role for metallothionein-2A against the growth-inhibitory effects of gallium nitrate is also suggested by experiments that show that zinc-induced upregulation of metallothionein diminishes the cytotoxicity of gallium in CCRF-CEM cells and that this protection decreases as metallothionein levels return to baseline [83].

Additional cellular processes may be altered by gallium. Protein tyrosine phosphatase inhibition by gallium nitrate has been demonstrated in Jurkat and HT-29 human colon cancer cells, but a correlation between enzyme inhibition and cell proliferation was not found [84]. Gallium can inhibit magnesium-dependent ATPase through competition with magnesium [85], and GaCl_3_ can inhibit tubulin polymerization, an effect that could contribute to its antineoplastic activity [86]. Early studies showed that DNA polymerases were partially inhibited by gallium nitrate; however, the extent of inhibition was insufficient to account for gallium’s antitumor activity [87]. In prostate cancer cell lines and a xenograft model, the proteasome was recently reported to be a target for the action of a new group of gallium compounds consisting of gallium complexed to ligands containing pyridine and 4-6-substituted phenolic moieties [88]. All these effects of gallium are of interest, and their contributions to the antineoplastic activity of gallium warrant further investigation.

#### 2.6.3. Drug resistance

Drug resistance to antineoplastic agents remains a major obstacle to the successful treatment of cancer. The basis for tumor cell resistance to gallium nitrate is not well understood, and CCRF-CEM lymphoma and HL60 human leukemia cell lines with an acquired resistance to gallium nitrate have been developed as tools to investigate the underlying mechanisms for this resistance. In HL60 cells, the development of resistance to growth inhibition by gallium nitrate is associated with a decrease in transferrin receptor-1 levels, a decrease in iron uptake, and an alteration in the intracellular trafficking of iron and gallium [89]. A downregulation of transferrin receptor-1 expression and gallium and iron uptake was seen in gallium-resistant CCRF-CEM cells [90,91]. The antiproliferative effect of gallium nitrate in these gallium-resistant cells could be in increased by increasing the concentration of exogenous transferrin in the culture medium [90,92]. These studies suggest that the development of gallium resistance includes changes in the uptake and intracellular transport of gallium. However, other processes may also be involved. Analysis of gallium-resistant and sensitive CCRF-CEM cells using a cDNA microarray approach that evaluated 96 genes associated with metal metabolism revealed a marked upregulation of metallothionein-2A, zinc transporter-1, and increased activity of metal responsive transcription factor-1 in cells resistant to gallium nitrate [83]. These studies suggest an action of gallium on proteins involved in zinc metabolism and a possible effect of metallothionein as a modulator of gallium sensitivity in certain cells. Metallothionein is variably expressed in different lymphomas *in vivo* [93]; therefore, a question to be addressed in future clinical trials is whether this expression affects the response of this malignancy to treatment with gallium compounds.

## 3. Some Future Directions for the Development of Gallium Compounds in Medicine

### 3.1. New Gallium Compounds with Antitumor Activity

Gallium nitrate is a simple metal salt that can be considered to belong to a first generation of gallium compounds. There is considerable interest in gallium-based metallodrugs and novel gallium agents are in development as illustrated in Table 1. These include the oral formulations gallium maltolate, G4544, and KP46, all of which are in clinical development [5,65,94]. Gallium maltolate inhibits hepatocellular carcinoma cell growth and induces apoptosis through action on the mitochondrial pathway in lymphoma cell lines [81,95]. On a mole-to-mole basis, this compound appears to be more cytotoxic to malignant cell lines *in vitro* than gallium nitrate. G4544 is being examined clinically for bone metastases and metabolic bone disease but may also prove to be active as an antineoplastic agent [65]. KP46 (Tris(8-quinolonato)Ga(III) is in clinical trials in Europe [96] and has been shown to induce apoptosis in a large panel of malignant cell lines [5,97]. In preclinical studies, gallium complexes of ligands containing pyridine and 4,6-substituted phenolate moieties with antitumor activity have been developed and have shown activity against prostate cancer xenografts in rats [88,98]. In addition, gallium thiosemicarbazones and novel organometallic gallium compounds are being aggressively advanced as therapeutic agents [99–102]. It is possible that these newer agents may have additional mechanisms of action that may be somewhat different from those of gallium nitrate. For example, gallium maltolate was recently shown to inhibit the growth of lymphoma cell lines with either acquired or inherent resistance to the cytotoxicity of gallium nitrate [81]. These data would suggest that the mechanisms of cell death induction by gallium maltolate occurs through pathways that are different than that for gallium nitrate [81]. The availability of lymphoma cell lines that are resistant to gallium nitrate-induced apoptosis provides the opportunity to examine whether the other newer gallium compounds share cross-resistance to gallium nitrate and whether these compounds may be more efficacious as therapeutic agents. The advancement of new gallium compounds to further preclinical and clinical testing is awaited with great anticipation.

### 3.2. Gallium Compounds as Antimicrobial Agents

The ability of gallium to interfere with the utilization of iron by certain microorganisms has led to some interesting investigations directed at exploring the potential of gallium as an antimicrobial drug. Gallium nitrate and transferrin-gallium were shown to block the iron-dependent growth of *Mycobacterium tuberculosis* and *M. avium* complex extracellularly as well as within human macrophages. Gallium interfered with iron acquisition by *M. tuberculosis* within the macrophage phagosome resulting in a bactericidal action which could be prevented by excess iron [108]. In another study, gallium nitrate inhibited *Pseudomonas aeruginosa* growth and biofilm formation, and killed planktonic and biofilm bacteria *in vitro* through mechanisms that included interference with iron uptake and iron signaling by the transcription regulator *pvdS*. In this study, gallium was effective against *P. aruginosa* pneumonia in mice and prolonged their survival [106]. In a mouse model of thermal injury inoculated with *P. aeruginosa*, gallium maltolate provided a 100% survival compared with 100% mortality in control mice not treated with gallium [107]. Patients with cystic fibrosis often develop *Pseudomonas* lung infections that may be resistant to standard antibiotics and difficult to eradicate; a clinical trial is in progress to assess the efficacy of gallium nitrate in the treatment of lung infections in this patient population (Genta Incorporated Inc; Press release December 2, 2009).

## 4. Challenges to Advancing Gallium Compounds as Therapeutic Agents in the Clinic

Gallium nitrate is presently approved for use in the clinic; however, for optimal efficacy, this drug needs to be administered by continuous intravenous infusion 24-hours a day for 5–7 days. This is obviously a cumbersome mode of treatment. It would be highly desirable to have gallium compounds that could be administered orally and have at least equivalent (or greater) therapeutic efficacy than intravenous gallium nitrate. Hence, there is a great need to continue to develop such agents, establish their therapeutic index (efficacy to toxicity ratio) in preclinical studies and advance them to human trials. At the same time, further research is needed to understand the molecular targets of gallium and its mechanisms of action. Clinical experience with gallium nitrate has shown that some patients with lymphoma resistant to conventional chemotherapy have responded dramatically to a single course of treatment whereas others have responded only partially or not at all [29,109]. This begs the question as to why certain cancers are inherently resistant to gallium while others are exquisitely sensitive. An understanding of the basis of drug resistance to gallium may allow us to use molecular markers in tumors to identify patients most likely to benefit from gallium-based treatment and to design newer gallium compounds that could bypass such resistance.

## 5. Toxicities of Gallium Compounds in Current Use

### 5.1. Gallium Nitrate

Treatment with continuous intravenous infusion gallium nitrate at the present recommended dose for hypercalcemia (200 mg/m^2^/day for 5 days) is generally well-tolerated, even by elderly patients. Higher continuous infusion doses (300 mg/m^2^/day for 5–7 days) are used for treatment of cancer. In phase 1 and 2 studies, renal toxicity was dose-limiting when gallium nitrate was administered as a brief intravenous infusion over 30 minutes [60]; this side-effect was seen in ∼12.5% of patients treated for hypercalcemia by continuous intravenous infusion. With the continuous infusion schedule for treatment of lymphoma, diarrhea rather than renal toxicity was dose-limiting [26]. Renal toxicity can be minimized by ensuring adequate fluid intake and avoiding of coadministration of nephrotoxic drugs. Microcytic anemia may develop in patients treated with gallium nitrate; however, platelet counts and white blood cell counts are not suppressed [23]. The latter is a major advantage as it means that unlike other chemotherapeutic drugs, gallium nitrate can be administered to patients with thrombocytopenia or neutropenia. Visual and auditory toxicities may occur but have been reported in <1% of patients. Hypocalcemia may develop in patients with normal blood calcium levels; this can be managed by oral supplementation with calcium carbonate.

### 5.2. Gallium Arsenide

Gallium arsenide is a valuable compound that is employed widely in the electronics industry. It possesses several advantages over silicon in that it has several-fold greater electron mobility and only a fraction of the electrical capacitance of silicon [110]. This property, along with its light-emitting, electromagnetic, and photovoltaic properties allow it to be used extensively in high-speed semi-conductor devices, in high power microwave and millimeter-wave devices, and in optoelectronic devices [1]. The latter include light-emitting diodes (LEDs), laser diodes, photodetectors and solar cells that are used in numerous applications.

Workers in the microelectronics industry may be exposed to gallium arsenide during the process of sandblasting gallium arsenide ingots, in the slicing, grinding, and polishing of gallium arsenide-silicon wafers, and in clean-up of work areas [1]. For these individuals, the primary portals of entry of gallium arsenide into the body would be via the respiratory tract and, to a much lesser extent, the gastrointestinal tract. Gallium and arsenic have been shown to be elevated in ground water obtained from semiconductor manufacturing industrial areas, thus suggesting that contaminated drinking water may also be a source of environmental exposure to gallium [111]. While it is felt that the toxicities of gallium arsenide are related primarily to the arsenic component of this compound, the contribution of gallium to some of these toxicities cannot be discounted especially in light of the fact that other gallium compounds display antineoplastic and antimicrobial activities. Therefore, a discussion of the toxicities of gallium arsenide and the relative contribution of gallium to these toxicities is warranted.

#### 5.2.1. Animal studies of gallium arsenide toxicity

An understanding of the potential risks of acute, subacute, or chronic environmental exposure to gallium arsenide has been gained through studies in which rats and mice were exposed to gallium arsenide under conditions of inhalation, intratracheal administration, or oral intake.

### Inhalation of gallium arsenide

The National Toxicology Program reported the effects inhalation of gallium arsenide particles by F344/N rats and B6C3F1 mice produced over a period of 16 days, 14 weeks, or 2 years [112]. In the 16-day study, animals were exposed to 0, 1, 10, 37, 75, or 150 mg/m^3^ gallium arsenide inhalation, 6 hours per day, 5 days per week for the duration of each period. At the end of the study, animal weights were similar to controls, and there were no deaths. The liver and lung weights were increased in male rats exposed to >1 mg/m^3^ and in female rats and mice of both sexes exposed to >10 mg/m^3^, while thymus weights were decreased in male rats exposed to 10 mg/m^3^. Gallium arsenide particles were detected in the alveolar spaces and within alveolar macrophages at higher concentrations in both animals. Minimal histiocytic cellular infiltrate and moderate proteinosis in the lung were noted in animals exposed to >10 mg/m^3^, while laryngeal epithelial hyperplasia and squamous metaplasia were noted in mice and rats exposed to >10 mg/m^3^ and 150 mg/m^3^ gallium arsenide, respectively. Mild chronic inflammation was noted in mice exposed to 75 or 150 mg/m^3^.

With a 14-week inhalation exposure, rats and mice were exposed to gallium arsenide particulate at concentrations of 0, 0.1, 1, 10, 37, or 75 mg/m^3^, 6 hours per day, 5 days per week for 14 weeks. No animal deaths were observed; however, final mean body weight and weight gain were significantly diminished in male animals compared with controls. A microcytic anemia with increased zinc protoporphyrin/heme ratios, increased platelet and neutrophil counts, and increased serum alanine aminotransferase and sorbitol dehydrogenase was noted. In males, testicular toxicity was noted at the higher concentrations of gallium arsenide that was characterized by a decrease in weights of the testes, cauda epididymis, and epididymis. Testicular atrophy was seen at the highest concentration of gallium arsenide in male rats, while total spermatid heads, spermatid counts, and spermatic motility were significantly decreased at lower concentrations. Increases in the incidence of plasma cell hyperplasia of mandibular lymph notes, bone marrow hyperplasia, and hemosiderosis of the liver and spleen were observed with the higher concentrations of gallium arsenide. The pulmonary toxicity noted with gallium arsenide inhalation for 14 weeks was an extension of that seen with a 16-day exposure. Lung weights were increased; proteinosis, histiocytic infiltration, and epithelial hyperplasia in the lung accompanied by inflammation and squamous metaplasia in the larynx were observed with the higher concentrations of gallium arsenide.

In a 2-year study of gallium arsenide inhalation exposure, B6C3F1 mice were exposed to 0, 0.1, 0.5, or 1.0 mg/m^3^ gallium arsenide for 6 hours a day, 5 days per week for 105 weeks (males) or 106 weeks (females). This resulted in a spectrum of inflammatory and proliferative lesions in the respiratory tract without the development of cancer. In another study, male and female Fischer 344/N rats were exposed to inhalation of 0–1 mg/m^3^ of gallium arsenide particles for 6 hours per day, 5 days a week for 105 weeks. Although there was no adverse impact on survival, inflammatory lesions, atypical hyperplasia, and metaplasia in the respiratory tract were noted. Particles of gallium arsenide were detected in the alveolar spaces and macrophages in animals exposed to the higher concentrations of this compound [112]. In addition to displaying a spectrum of such changes in the lung, female but not male rats had a gallium arsenide concentration-dependent increase in benign and malignant lung lesions (alveolar/bronchiolar, adeno, and squamous cell carcinomas). Females also had a gallium arsenide concentration-dependent increase in adrenal neoplasms, and an increased incidence of large granular lymphocytic leukemia with exposure to 1.0 mg/m^3^ of this compound.

### Intratracheal administration of gallium arsenide

The toxicities of gallium arsenide administered by intratracheal installation to rodents using various schedules ranging from a single dose to repetitive exposure are summarized in the studies in Table 2. Details of these and related studies have been presented in recent reviews [1,113]. As with inhalation exposure, intratracheal installation of gallium arsenide produces various levels of damage to the respiratory tract. In addition, as shown in Table 2, this mode of gallium arsenide delivery may also produce testicular toxicity, inhibition of the heme biosynthetic pathway, decreases in hemoglobin levels, renal damage, and suppression of immunological functions.

### Oral administration of gallium arsenide

Two representative studies of oral gallium arsenide in animals are summarized in Table 3. With oral administration, the majority of gallium and arsenide appears in the feces thus indicating that this compound has limited bioavailability. Not surprisingly, pulmonary toxicity is not encountered with oral gallium arsenide; however, Flora *et al.* (Table 3) found a dose-dependent inhibitory effect of oral gallium arsenide on heme biosynthesis and primary immune response along with evidence of hepatic oxidative stress [125]. Collectively, animal studies show that gallium arsenide administered by inhalation or by intratracheal instillation is considerably more toxic than oral gallium arsenide.

#### 5.2.2. Human studies

Several studies have reported on the exposure of workers to gallium arsenide and other metals in the optoelectronic industry in Taiwan. Liao *et al.* measured gallium and arsenic levels in the blood and urine of 4 separate groups of workers: (1) fabrication equipment preventative maintenance workers, (2) dopants and thin film (dope film) workers, (3) fabrication supervisors and engineers, and (4) nonexposed office workers. Significantly increased levels of gallium and arsenic were detected in the urine of the first three groups of workers exposed to gallium arsenide compared with the non-exposed control group [126]. The use of masks and gloves had a protective effect against gallium arsenide exposure indicating that this compound may be taken up by workers through the respiratory tract and skin [126]. In another study, Liao *et al.* showed that blood and urine levels of malondialdehyde (MDA), the byproduct of lipid peroxidation, was elevated in electronics industry workers exposed to aluminum, gallium, indium, and arsenic and that MDA levels strongly correlated with urinary levels of gallium and arsenic [127]. In an additional investigation, Chen measured metal concentrations in the inhalable air and in the urine of workers in the workplace of two major semiconductor companies in Taiwan [128]. The gallium concentration in inhalable air samples from the work areas of production operators and engineers (exposed groups) ranged from 0.34–101.26 μg/m^3^ while the urinary gallium levels ranged from 4.42–56.31 μg/L [128]. In contrast, gallium levels in the inhalable air and in the urine of control, unexposed individuals (office administrators) were 0.14–18.0 μg/m^3^ and 0.09–8.05 μg/L, respectively [128].

### 5.3. What is the Contribution of Gallium to the Toxicities of Gallium Arsenide?

In aqueous medium, gallium arsenide rapidly dissociates to form gallium and arsenic oxides which may be further hydrolyzed [114]. The toxicities of arsenic are well recognized, and it has been suggested that the toxicities of gallium arsenide may be largely caused by the arsenic component of this compound [129]. However, this may be dependent on the target organ examined and on the conditions under which animal experiments were conducted. The following studies shed some light on this matter.

#### 5.3.1 Pulmonary toxicity

Webb *et al.* compared the pulmonary toxicity of a single intratracheal administration of 100 mg/kg gallium arsenide with that of gallium trioxide (Ga_2_O_3_) and arsenic oxide (As_2_O_3_) in male Fischer-344 rats [115]. Histopathological changes of inflammation, necrosis, neutrophil infiltration, and fibrosis were noted in the animals treated with gallium arsenide and As_2_O_3_. In contrast, Ga_2_O_3_-treated animals displayed an increase in lipid and only mild pneumomonocyte hyperplasia. Visible particles of Ga_2_O_3_, nodules of macrophages, and phagocytosis of these Ga_2_O_3_ particles by macrophages were also seen in these lungs. Hence, gallium does not appear to be a pneumotoxicant.

#### 5.3.2. Heme synthesis

Intratracheal administration of gallium arsenide to rats results in the suppression of the heme synthesis pathway via inhibition of delta-aminolevulinic acid dehydratase (ALAD) in several tissues [117]. In a separate study that examined the effects of gallium independent of arsenic, Goering and Rehm showed that within 24 hours of administration, a single intraperitoneal dose of soluble gallium sulfate (12.5–200 mg Ga/kg) produced a dose-dependent noncompetitive inhibition of ALAD in the liver, kidney, and erythrocytes (inhibition constant for ALAD was approximately 3 μM gallium) [130]. Interestingly, the inhibitory effects of gallium on hepatic and renal ALAD was attenuated by zinc suggesting that gallium’s action on this enzyme may involve displacement of zinc from its binding to the active sulfhydryl group of the enzyme [130]. This study clearly indicates that gallium per se can inhibit ALAD and may thus contribute to the inhibitory effect of gallium arsenide on heme synthesis; however, an important factor determining this effect may be the route of entry of gallium into the body.

#### 5.3.3. Testicular toxicity

The available data on the effects of gallium on the testes are conflicting. Omura *et al.* compared the testicular toxicities of gallium arsenide, indium arsenide, and As_2_O_3_ in male Wistar rats receiving these compounds by repetitive intratracheal instillation twice a week for 16 treatments [120]. A significant increase in morphologically abnormal sperm, a decrease in sperm count, and an increase in degenerating late spermatids were seen in the gallium arsenide-treated animals. Indium arsenide showed much weaker testicular toxicity than gallium arsenide while no toxicity was seen with As_2_O_3_. It was concluded that gallium likely contributed to the testicular toxicity of gallium arsenide [120]. In contrast, Colombina *et al.* administered increasing doses of gallium nitrate to male mice subcutaneously every other day for 14 days and did not see an effect of gallium on fertility, reproduction, sperm counts, and the testes [131]. Unfortunately, there are only a limited number of studies examining the effect of gallium per se on the testes, so that firm conclusions regarding this toxicity of gallium need to be made with caution. But, it is important to note that there were differences in the schedule and mode of administration of gallium (intratracheal *versus* subcutaneous injection and 8 weeks *versus* 14 days) in these two studies. Thus, the possibility that prolonged, chronic exposure to gallium may produce testicular damage cannot be excluded.

#### 5.3.4. Immunosuppression

An important toxicity of gallium arsenide is its suppressive action on the immune system. Sikorski *et al.* compared the effects of gallium arsenide and sodium arsenite on cellular and humoral responses in female B6C3F1 mice 14 days after the administration of a single intratracheal installation of either agent [132]. A differential effect of gallium arsenide and sodium arsenite on immune parameters was noted. The splenic IgM response to the T-dependent antigen sheep red blood cells was reduced by gallium arsenide in a dose-dependent manner to a 65.5% decrease at the highest dose of this compound (200 mg/kg); this was accompanied by a significant decrease in spleen weight and cellularity. In contrast, sodium arsenite decreased the splenic IgM response by 24% and did not affect spleen size or cellularity. The spleen cells from gallium arsenide-treated mice displayed a dose-related suppression of the mixed lymphocyte response (MLR) to the presence of allogeneic stimulating cells; in contrast, sodium arsenite-treatment not did result in suppression of the MLR [132]. Natural killer cell activity was increased in gallium arsenide-treated but not in sodium arsenite-treated mice. Gallium arsenide treatment but not sodium arsenite treatment appeared to increase the resistance of mice to mortality from Listeria monocytogenes infection. However, gallium arsenide but not sodium arsenite decreased the resistance of mice to the growth of implanted B16F10 melanoma cells [132]. Collectively, these studies indicate that the effects of gallium arsenide on the immune system are not due to arsenic alone and that gallium contributes significantly to the action of this compound.

In a separate study however, Burns *et al.* examined the individual roles of gallium and arsenic on the immunosuppressive activity of gallium arsenide by measuring the primary antibody-forming cell (AFC) response of B6C3F1 mice splenocytes to sheep erythrocytes *in vitro* [133]. Suppression of the *in vitro* AFC IgM response was seen with gallium arsenide, gallium nitrate, and sodium arsenite. However, the authors concluded that arsenic was the primary contributor to the early immunosuppressive effects of gallium arsenide based on the observations that: (1) the concentration of gallium required to inhibit the early IgM response was greater when gallium was added to the incubation as gallium nitrate than when it was added as gallium arsenide and, (2) the gallium chelator oxalic acid did not reverse gallium arsenide-induced IgM response [133].

Considering the results of other studies that demonstrate that gallium compounds other than gallium arsenide display can suppress the immune system (discussed in Section 2.4), the collective data would indicate that gallium per se also contributes to the immunosuppressive action of gallium arsenide.

## 6. Conclusions

Almost 135 years after its discovery, gallium continues to show promise for the treatment of certain diseases. However, this potential needs to be further explored and the newer gallium compounds being developed should be advanced to clinical trials after rigorous preclinical testing. At the same time, there needs to be a better understanding of gallium’s mechanisms of antineoplastic activity and its molecular targets. It is envisioned at such information would enable us to identify diseases and patients who would selectively benefit from gallium-based therapy.

## Figures and Tables

**Table 1.** Gallium Compounds in Clinical and Preclinical Development.

Gallium Compounds	Tumors or diseases investigated/being investigated
First Generation–FDA-approved	
Gallium nitrate [103–106].	Hypercalcemia, metabolic bone disease Bladder cancer, lymphoma, other cancers Microbial infections
Second generation–Preclinical and Phase 1 and 2 clinical trials	
Gallium maltolate [81,95,107]	Hepatoma, lymphoma, microbial infections
G4544 [65]	Metabolic bone disease, osteoporosis, skeletal metastases
Tris(8-quinolonato)Ga(III) KP46 [5;96;97]	Lung cancer, melanoma, other cancers
Third generation–Preclinical	
Gallium thiosemicarbazones [99–101]	Various malignant cell lines, cryptococcus fungi
Gallium complexes with ligands of pyridine & 4–6-substituted phenolic moieties [88]	Prostate cancer (rat model)
Organometallic gallium compounds [102]	Various malignant cell lines

**Table 2.** Toxicities of Intratracheal Installation of Gallium Arsenide (GaAs) in Animals.

Organ systems and animals	Outcome
Pulmonary	
Male Fischer-344 rats; single installation of 10, 30, or 100 mg/kg GaAs; analysis at 14 days [114]	Weight loss; dose-dependent increase in lung weight; 14–42% of As and 23–42% of gallium dose retained in lung; arsenic, but not gallium detected in the blood; increased urinary excretion of porphyrins
Male Fischer-344 rats; single installation of 100 mg/kg GaAs; analysis at 14 days [115]	Increased weight and dry lung weight with elevation of lung protein, DNA, and 4-hydroxyproline; pulmonary retention of GaAs particles with multifocal alveolitis, purulent pneumonia, Type II pneumocyte hyperplasia, necrosis, and mild fibrosis
Male Fischer-344 rats; single installation of smaller particle diameter “respirable” 100 mg/kg GaAs. Analysis on days 1, 3, 7, 14, and 28 [116]	Loss of body weight, pneumocyte hyperplasia, proliferative pneumonia, interstitial pneumonia, perivascular cuffing, lymphoid hyperplasia, edema, fibrosis, hemorrhage, vascular congestion, and alveolar proteinosis
Male CD rats; single installation of 50, 100, or 200 mg/kg GaAs [117]	Increased lung weight, and seropurulent pneumomia; increase in Type II pneumocytes and alveolar macrophases; interstitial pneumonia
Syrian golden hamsters; Installation of 0.5 mg GaAs per week for 15 weeks, 2 year observation [118]	Reduced survival and pulmonary inflammation
Syrian Golden Hamsters; 7.6 mg/kg GaAs twice per week x 4 weeks. Evaluated at 8 and 16 weeks [113]	Moderate inflammation with diffuse alveolar bronchiolar cell hyperplasia and infiltration with alveolar macrophages; mild interstitial fibrosis and cholesterol clefts
Testicular	
Syrian Golden hamsters; 7.7 mg/kg GaAs, twice a week for 14 installations [119] Rats; 7.7 mg/kg GaAs, twice a week for 14 installations [120]	Sperm count reduced by 22%, 3-fold increase in spermatid retention and degeneration, increase in abnormal sperm Sperm count reduced by 41%; 40-fold increase in degenerating late elongated spermatids
Renal	
Male CD rats; single installation of 50, 100, or 200 mg/kg GaAs [117]	Mitochondrial swelling in proximal tubules
Syrian Golden Hamsters [113]	Atrophy and degenerative changes of convoluted tubules
Heme biosynthesis	
Male CD rats; single intratracheal installation of 50, 100, or 200 mg/kg GaAs [117]	Dose-dependent inhibition of blood delta-aminolevulinic acid dehydratase (ALAD) in erythrocytes (by 3 days), and kidney (by 6 days), increased urinary ALA excretion by 6 days
Immunologic	
B6C3F1 mice; single intratracheal dose of 50–200 mg/kg GaAs [121–124].	Suppression of both humoral and cell-mediated immunity; inhibition of T-cell proliferation; altered CD25 expression; suppression of macrophage processing of antigens and IgM response

**Table 3.** Toxicities of Orally Administered Gallium Arsenide in Animals.

Animals	Outcome
Male Fischer-344 rats Single oral dose of 10, 100, or 1000 mg/kg GaAs; analysis at 14 days [114].	Dose-dependent recovery of gallium (70–99%) and arsenic (56–91%) in the feces. Arsenic, but not gallium, detected in the blood. Significant increase in urinary uroporphyrin excretion with highest dose. No pathologic changes in lungs, liver, spleen, kidney, or testes.
Male Wistar albino rats Single oral dose of 10, 200, or 500 mg/kg GaAs. Analysis at 1, 7, and 21 days [125].	Weight loss. Increased liver weight, increased serum gamma-glutamyltranspeptidase and serum aspartate aminotransferase activity, decreased hepatic malondialdhyde and glutathione content. Increased renal alkaline phosphatase activity. Decreased erythrocyte delta-aminolevulinic acid dehydratase (ALAD); increased urinary ALA and protein excretion. Dose-dependent decrease in spleen weight and cellularity, and decreased IgM production by antibody-forming cells in response to sheep erythrocytes.
